# Understanding the Role of T-Cells in the Antimyeloma Effect of Immunomodulatory Drugs

**DOI:** 10.3389/fimmu.2021.632399

**Published:** 2021-03-05

**Authors:** Criselle D'Souza, H. Miles Prince, Paul J. Neeson

**Affiliations:** ^1^Cancer Immunology Program, Peter MacCallum Cancer Centre, Melbourne, VIC, Australia; ^2^Sir Peter MacCallum Department of Oncology, University of Melbourne, Melbourne, VIC, Australia; ^3^Clinical Hematology, Peter MacCallum Cancer Centre and Royal Melbourne Hospital, Melbourne, VIC, Australia

**Keywords:** immunomodulatory drugs, myeloma, T cells, NK cells, immunotherapy

## Abstract

Immunomodulatory drugs (IMiDs) are effective treatments for patients with multiple myeloma. IMiDs have pleotropic effects including targeting the myeloma cells directly, and improving the anti-myeloma immune response. In the absence of myeloma cells, lenalidomide and pomalidomide induce CD4^+^ T cell secretion of IL-2 and indirect activation of Natural Killer (NK) cells. In the context of T cell receptor ligation, IMiDs enhance T cell proliferation, cytokine release and Th1 responses, both *in vivo* and *in vitro*. Furthermore, combination treatment of IMiDs and myeloma-targeting monoclonal antibodies eg. daratumumab (anti-CD38) and elotuzumab (anti-SLAMF7), checkpoint inhibitors, or bispecific T cell engagers showed synergistic effects, mainly via enhanced T and NK cell dependent cellular toxicity and T cell proliferation. Conversely, the corticosteroid dexamethasone can impair the immune modulatory effects of IMiDs, indicating that careful choice of myeloma drugs in combination with IMiDs is key for the best anti-myeloma therapeutic efficacy. This review presents an overview of the role for T cells in the overall anti-myeloma effects of immunomodulatory drugs.

## Introduction

Multiple myeloma (MM) is a plasma B cell malignancy primarily localized to the bone marrow and characterized by immune dysfunction due to the complex interplay between the malignant plasma cells (PC) and immune cells in the tumor microenvironment (1). New therapeutic approaches in the last two decades have dramatically improved patient outcomes. These include the incorporation of immunomodulatory drugs (IMiDs) that exert pleiotropic effects including directly acting on the malignant cells and enhancing T and NK cell anti-myeloma properties (2).

## Immunomodulatory Drugs

IMiDs, namely lenalidomide and pomalidomide are a group of drugs that are derivatives from thalidomide, a glutamic acid derivative (2). Thalidomide use in clinical trials of advanced MM was first published in 1999 and soon it was utilized, alone and in combination, across all phases of myeloma therapy (3). Due to its problematic side effects and improved efficacy of the subsequent IMiDs, thalidomide has progressively been replaced by lenalidomide and pomalidomide in the myeloma treatment paradigm; currently IMiDs are used in newly diagnosed MM (NDMM) patients, in maintenance therapy post autologous stem cell transplantation and in patients with relapsed refractory multiple myeloma (RRMM) (4).

IMiDs were first shown to have anti-angiogenic activities and anti-inflammatory anti-tumor necrosis factor (TNF)-α activity in monocytes (5). Although, IMiDs suppress TNFα production by monocytes (5), they have an opposite effect on T cells, where they increase TNFα production (6). IMiDs also directly act on myeloma cells, affecting their proliferative capacity (7).

## Mechanism of Action

IMiDs bind to Cereblon (CRBN), DDB1, CUL4 and ROC1 and together form an E3 ubiquitin ligase, which modify multiple proteins causing ubiquitination and proteasome degradation of these target proteins (8). IMiDs cause selective degradation and downregulation of two CRBN-binding lymphocyte transcription factors IKZF1 (Ikaros) and IKZF3 (Aiolos), which leads to an increase in interleukin (IL)-2 production in T cells (8) and mechanism reviewed in detail in (9). In addition, IMiDs can also cause immune activation by stimulating NK cells, and inhibiting IL-6 production from monocytes and macrophages (10, 11).

CRBN-binding protein Casein kinase 1 alpha (CK1a) is degraded upon IMiD treatment. CK1a is known to facilitate MM pathogenesis as it sustains activation of oncogenic cascades through p53 activation, phosphoinositide-3-kinase (PI3K) / protein kinase B (AKT) and nuclear factor kappa B (NF-κB) activation and modulation of interferon (IFN) pathway (12, 13).

## Multiple Myeloma Development Leads to Immune Dysfunction

Several studies have shown that myeloma cell survival and immune escape is facilitated by impaired endogenous signaling in immune cells (2, 14). These include defects in T cell distribution and function, a reduction of peripheral blood CD4^+^ and CD8^+^ T cells, abnormal Th1/Th2 ratio, decrease in the CD4/CD8 T cell ratio with reduced or aberrant T cell function, and reduction in NKT cells (14). Myeloma cells are known to secrete transforming growth factor (TGF)-β, a highly immunosuppressive cytokine that inhibits T and NK cell function and cytotoxicity. The compartment in which these cells are residing/trafficking may be relevant. Indeed, a recent study demonstrated that at the tumor site within the bone marrow, T cells are immune-suppressed, largely exhausted and senescent (15).

MM production of TGF-β, IL-10, IL-6, and VEGF also leads to impaired DC antigen presentation to T cells, affecting T cell priming (16, 17). Myeloid and plasmacytoid dendritic cells (DCs) from MM patients have lower expression of human leucocyte antigen (HLA) molecules, C-C chemokine receptor (CCR)-5, CCR7, and DEC205 (18). This further leads to a suboptimal immune response toward tumor cells. Some reports suggest regulatory T cells (Tregs), a subset of immune-suppressive CD4^+^ T cells, to be increased in MM patients (19, 20). However, this is controversial as there are also conflicting reports with lower numbers of Tregs in patients than healthy controls (21). These discrepancies could be due to technical issues with absolute counts and percentages of cells, if particular subsets of CD4^+^ T cells are reduced. This could also be related to dexamethasone treatment. There is also a skewing of Treg/Th17 balance in MM and this further increases immune suppression leading to poor prognosis in patients (22, 23). Overall, immune evasion of tumor cells is associated with myeloma cell proliferation and leads to MM pathogenesis. Treatments that can reverse these defects in combination with other treatments such as monoclonal antibodies have shown the most promise in treating MM in currently approved therapies.

## Immune Modulation by IMiDs

*In vitro* studies have shown that treatment with IMiDs enhanced T cell proliferation, IL-2 and IFN–γ secretion and NK and NKT cell activation (24, 25). IMiDs such as thalidomide downregulated IL-6 from peripheral blood mononuclear cells (PBMC) and IL-10 production from T cells and induced co-stimulation of CD4^+^ and CD8^+^ T cells in *in-vitro* assays (10). Pomalidomide and lenalidomide are 300–1,200 times more potent than thalidomide at inducing T cell proliferation, IL-2 and IFN-γ production (10, 24). IMiDs enhanced DC-antigen presentation leading to activation of CD8^+^ and CD4^+^ T cells and production of IFN-γ (26, 27). IMiDs also stimulated CD28 downstream signaling by binding to B7 co-stimulatory molecule, reducing myeloma immune tolerance (28). IMiDs enhanced expression of DNA-binding protein AP-1, which in turn causes CD28 signaling and stimulation of nuclear factor of activated T-cells (NFAT) (2, 28, 29). This leads to production of IL-2 inducing T cell proliferation and activation and also NK cell activation (29).

*In vivo*, IMiDs were shown to increase endogenous tumor-specific T cell and NK cell immunity as well as in vaccine responses (30).

## Cytotoxic CD8^+^ T Cells

Studies have shown that central and effector memory CD8^+^ T cells, Tregs and myeloid derived suppressor cells (MDSC) increased after lenalidomide treatment (31). Thus, indicating that lenalidomide treatment can induce both activating and inhibitory immune responses. However, the inhibitory effects observed could be due to dexamethasone (an immunosuppressant) combination treatment that was used for some patients in this study. Indeed, we have previously showed that high dose dexamethasone abrogates immune activating effects of lenalidomide (32).

Myeloma patients that received bortezomib treatment followed by lenalidomide maintenance treatment post autologous stem cell transplant (ASCT) showed increase in naïve and memory CD8^+^ T cells, higher expression of co-stimulatory molecules and reduction in inhibitory checkpoint molecules (33). However, an increase in Treg cells was also observed in this study. Anti-myeloma specific T cell responses with increased secretion of IFN-γ, perforin and granzyme B were observed in a clinical trial of myeloma patients that received lenalidomide as consolidation therapy after ASCT (34).

In newly diagnosed patients, lenalidomide treatment after ASCT impaired long-term thymic T cell reconstitution, with a decrease in CD4^+^ and CD8^+^ effector T cell counts (6–18 months post graft) and increase in Tregs (9–18 months post graft) (33, 35). Lenalidomide maintenance treatment also reduced programmed cell death protein 1 (PD-1) expression on CD8^+^ T cells, suggesting that it has the potential to counter-act the myeloma induced exhaustion or senescence on T cells (36). However, an alternate explanation is that lenalidomide maintenance treatment maintains low minimal residual disease, leading to lower antigen stimulation and reduced T cell exhaustion.

## CD4^+^ T Helper Cells and Treg Cells

In the context of ASCT, IMiDs (with dexamethasone and bortezimib) treatment during induction chemotherapy has been shown to play a role in reducing the pro-tumor Th17-Th1 and Th22 cells in the bone marrow, concurrently with reduction in cytokine levels of IL-17, IL-22, and IL-6, TNF-α, IL-1β, and IL-23 (37). This correlated with a favorable clinical outcome (37). There was no difference in the number of Tregs between treated and untreated or between diagnosis and transplantation in this study (37). However, previous studies showed an increase or decrease in Treg numbers in patients treated with lenalidomide and dexamethasone (38–40). In *in-vitro* assays, lenalidomide and pomalidomide inhibited IL-2 mediated generation of Tregs from PBMCs with marked reduction in suppressor function (41). In a post-transplant setting, peripheral blood Tregs declined in patients treated with IMiDs during induction therapy pre-ASCT as CD8 T cells expanded (38). In contrast, another study showed that Treg numbers increased in relapsed patients when treated with lenalidomide post allogeneic stem cell transplant (allo-SCT) and with 46% of the patients responding to the therapy (39). Thus, further highlighting the discrepancies observed in different studies on the role of IMiDs on Tregs.

## Effect of IMiDs on NK and Invariant Natural Killer T Cells

NK cells play an important role in tumor immunity. However, they are dysfunctional in myeloma (1). The immune suppressive myeloma microenvironment has elevated IL-10 and TGF-beta, and was associated with decreased expression of NK activating receptors, TNF and IFN-γ secretion, and impaired NK cytotoxicity toward myeloma (1, 42, 43).

Multiple studies demonstrated IMiDs enhanced the activity and function of NK and invariant NKT cells in multiple myeloma (24, 29, 44, 45). Lenalidomide enhanced direct NK cell cytotoxicity and NK-dependent antibody dependent cellular cytotoxicity (ADCC) (24). However, our study showed that combination treatment with high dose dexamethasone abrogates this lenalidomide induced NK cell function (32). Lenalidomide enhances NK cell cytotoxicity by CD4^+^ T cell production of IL-2 and dexamethasone suppresses this IL-2 production (32).

Combination treatment of IMiDs and monoclonal antibodies elotuzumab (anti-SLAMF7) or daratumumab (anti-CD38) and isatuximab (anti-CD38) showed synergistic effects in enhancing NK cell activity and efficacy in clinical trials (46–50). This is through enhancing NK ADCC, monocyte/macrophage mediated antibody dependent cellular phagocytocis (ADCP) and apoptosis (1). In phase 1/2 study of combination therapy of daratumumab, lenalidomide and dexamethasone in refractory relapsed MM patients, an overall response rate of 81% was achieved (51). In a phase 3 trial, patients that received the daratumumab, lenalidomide and dexamethasone, a higher overall response and progression free survival rate as compared to the lenalidomide and dexamethasone group (49). In phase 1 and phase II studies, patients with RRMM that received combination of elotuzumab, lenalidomide and dexamethasone had a 30% reduction in disease progression or death without significant toxicity (52, 53). These treatment strategies demonstrate that combining IMiDs with monoclonal antibodies are effective for RRMM.

Invariant NKT cells are CD1d restricted T cells that recognize glycolipid antigens. Invariant NKT cells respond to α-galactosylceramide (NKT cell antigen) pulsed primary myeloma cells, with release of cytokines and tumor cell lysis (54). MM patients treated with lenalidomide showed increased invariant NKT cell frequency with cytokine responses. Patients with refractory disease have a marked decrease in invariant NKT cell frequency (45). However, no difference in invariant NKT cell numbers were observed in newly diagnosed MM patients (45). Lenalidomide induction or maintenance therapy did not seem to have any effect on invariant NKT cell frequency and numbers (45). Thus, suggesting that lenalidomide benefits are not dependent on invariant NKT cells.

## Impact of Combination Treatment With IMiDs on T Cells

Immune checkpoint molecules such as cytotoxic T-lymphocyte-associated protein 4 (CTLA-4), PD-1 and lymphocyte activation gene 3 (LAG-3) diminish T cell responses and play a major role in peripheral tolerance to antigens and preventing autoimmune disease. In myeloma, PD-1 is expressed on bone marrow and peripheral blood CD8^+^ T cells and NK cells (55, 56). The PD-1 ligand (PD-L1) is expressed on malignant myeloma cells, bone marrow MDSC and PD-L1 is upregulated on interaction with bone marrow stromal cells (BMSC) (57, 58). Thus, indicating that the PD1-PDL1 axis can be targeted as a treatment strategy.

In *in-vitro* studies, single blockade using an antibody against PD-1 or dual blockade using antibodies against PD1 and PD-L1 with lenalidomide induced anti-myeloma immune response by blocking the cross-talk between BMSC and myeloma cells, thus reducing myeloma growth (55). A phase 1 trial of pembrolizumab (anti-PD-1 antibody) in combination with lenalidomide and dexamethasone showed a 50% overall response rate (59). A phase 2 trial with the same combination showed an overall response rate of 60% in refractory relapsed patients (60). However, a phase 3 trial (KEYNOTE-183 and KEYNOTE-185) evaluating pembrolizmab with lenalidomide and dexamethasone in newly diagnosed patients was discontinued due to unexpected higher risk of death (61). Other ongoing trials involving immune checkpoint inhibitors in NDMM patients were stopped based on this warning (62). Therefore, despite some trials showing a safe response, in light of the phase 3 trials described above, the safety concerns of the PD1/PDL1 inhibitors in combination with IMiDs led the FDA to terminate all trials using these combinations [reviewed in detail (62)].

A vaccine approach that used DCs fused with tumor cells demonstrated an increase in myeloma specific T cells, in a phase II study in the post ASCT setting (63). Combining IMiDs with this vaccine may further enhance the response to the DC fusion vaccine therapy. Indeed, combination treatment of a vaccine targeting the EGFR pathway substrate (Eps8) antigen on myeloma cells with lenalidomide enhanced the tumor-specific cytotoxic T cell response and increased survival in patients (64).

## IMiDs Effects on the Tumor Microenvironment and Myeloma Cells

The BMSC and the extracellular matrix (ECM) proteins such as CD44, VLA-4, LFA-1, VCAM, NCAM, and ICAM-1 are critical for the malignant PC survival (65). The interaction between the BMSC and PC is via cell-cell contact and production of cytokines such as IL-6, which promote PC survival. Adhesion of MM cells to BMSC enhances expression of IL-6 and VEGF (66). IMiDs inhibit production of TNFα, which in turn reduces the production of IL-6 (67). IMiDs downregulate surface adhesion molecules, which inhibit the MM-BMSC interaction and reduce the pro-survival cytokine production (67). IMiDs also exert direct effects on PC proliferation via inhibition of the cyclin-dependent kinase pathway and activation of Fas-mediated cell death (2).

**Figure 1:**
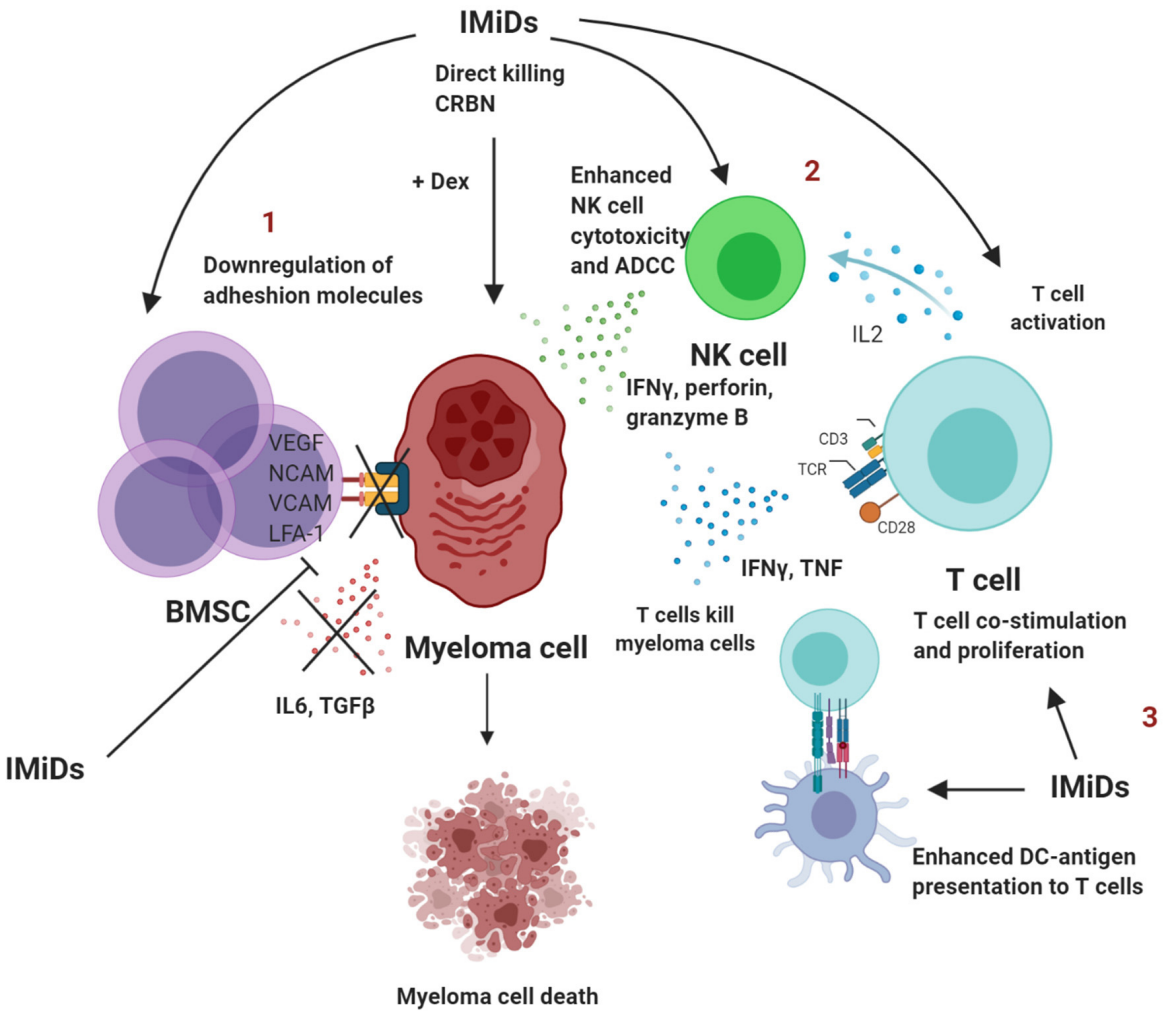
Summary of immunomodulatory effects of IMiDs in multiple myeloma. 1. IMiDs directly act by downregulating adhesion molecules that disrupt the BMSC-myeloma interaction, which further leads to reduced production of IL-6 and TGFβ and conversion to a less immune-suppressed TME. 2. IMiDs enhance IL-2 production by CD4^+^ T cells that in turn lead to NK cell activation and proliferation. IMiDs also enhance direct cellular cytotoxicity and ADCC. 3. IMiDs enhance DC-T cell antigen presentation and co-stimulation through the CD28 pathway. T cells secrete higher amounts of IL-2, IFNγ and TNF. BMSC, bone marrow stromal cells; DC, dendritic cells; NK, Natural killer; IL, interleukin; TGF, transforming growth factor; ADCC, antibody-dependent cellular toxicity; dex, dexamethasone; IFN, Interferon; TNF, tumor necrosis factor. Created with BioRender.com.

The inhibition of PC survival by IMiDs restores immune homeostasis and thus reduces the presence of senescent or exhausted T cells in the bone marrow. A summary of the immunomodulatory effects of IMiDs are outlined in Figure 1.

## Resistance to IMiDs

Treatment with IMiDs has improved survival of MM patients and is now a standard of care treatment (68). However, a subset of patients relapse and are refractory to IMiDs over time (69). One of the mechanisms that has been identified is through the CRBN pathway, which is a known primary target of IMiDs mediated degradation and ubiquitination as explained earlier (70). High expression of CRBN has been associated with improved clinical response in IMiD treated patients (70, 71). Patients with lenalidomide resistance express lower levels of CRBN than lenalidomide sensitive patients (70, 71).

A recent study has reported that RUNX transcription factor proteins, RUNX1 and RUNX3 interact with the transcription factor proteins, IKZF1 and IKZF3 (substrates for CRBN binding), which protects them from CRBN-dependent degradation that is induced by IMiDs (72). Other signaling pathways have been identified for IMiD resistance in myeloma cells. These include the Wnt/β-catenin (73), MEK/ERK (74), or STAT3 pathways (75). Overall, all the pathways identified for resistance to IMiDs are in myeloma cells. This may in turn affect the micro-environment leading to immune dysregulation. However, further studies are required to demonstrate whether IMiD resistance in myeloma cells, affects their capacity to inhibit T and NK cell function.

## Overcoming Resistance to IMiDs

New IMiDs known as CELMoDs (CRBN modulating agents) have been recently developed; these drugs have a more specific activity than IMiDs and are engineered to target specific proteins for rapid and efficient degradation (76). These CELMoDs can be used in patients that are refractory to previous lines of treatment that include lenalidomide and pomalidomide (76). These new CELMoDs include avodomide (CC-122) and iberdomide (CC-220) (77, 78). Avadomide has shown acceptable safety and pharmacokinetics in myeloma patients (78). Iberdomide is an E3 ligase cereblon modulator that mediates anti-proliferative and immunostimulatory activity in lenalidomide and pomalidomide resistant cell lines (79). Combination of iberdomide with daratumumab showed superior cytotoxicity against myeloma cell lines than either drug alone (79). In early results of an ongoing phase 1/2 clinical trial (NCT02773030), iberdomide plus dexamethasone showed favorable efficacy and safety in pre-treated RRMM patients who failed previous treatments, including pomalidomide, lenalidomide, and daratumumab (77). In other arms of the trial, iberdomide plus daratumumab plus dexamethasone (IberDd) and iberdomide plus bortezimib plus dexamethasone (IberVd), showed favorable tolerability with an overall response rate of 35% across both groups (80). These results demonstrate that triplet combination therapy may have clinical benefits in heavily pre-treated refractory patients. Further phase 3 trials are needed to evaluate these triplet combinations.

Other strategies have been tested to overcome IMiD resistance in *in-vitro* assays against myeloma cell lines resistant to IMiDs. These include STAT-3 inhibitor (PB-1-102) and MEK1/2 inhibitor, demonstrating the importance of these pathways in resistance to IMiDs (75). As RUNX proteins protect IKZF1 and IKZF3 from degradation, inhibition of RUNX resulted in sensitization of myeloma cell lines and primary tumors resistant to lenalidomide (72). Epigenetic modulators such as 5-Azacytidine (DNA methyltransferase inhibitor) and EZH2 inhibitor re-sensitized IMiD-resistant myeloma cells through extensive epigenetic reprogramming and independent of CRBN (81). However, the exact mechanism is still unknown.

## Conclusion

Treatment with IMiDs, especially in combination with other therapeutic drugs has dramatically improved the outcomes of patients with MM, both for newly diagnosed and refractory relapsed patients. IMiDs exert their action through multiple pathways. Immune modulation by enhancing the function of immune cells such as T and NK cells are a major pathway of their mode of action.

Newer therapies such as bispecific antibodies and chimeric antigen receptor (CAR)-T cell therapies are currently in clinical trials and are showing promising results in treating myeloma [reviewed in (82, 83)]. Both of these approaches target specific antigens on myeloma cells and work through CD3 stimulation (bispecific antibodies) or CAR on T cells, but independent of their T cell receptor (82, 83). Both of these therapies use T cells from myeloma patients, which are senescent or enriched in Tregs (as discussed in previous sections). IMiDs can reverse some of these defects in the T cells and restore immune homeostasis. Therefore, future therapeutic approaches could involve combining IMiDs with CAR-T cell based approaches and bispecific engagers, reducing chances of relapse. Overall, combination approaches will avoid drug resistance that has been observed with single agents.

## Author Contributions

CD'S, HP, and PN co-wrote and edited the manuscript. All authors contributed to the article and approved the submitted version.

## Conflict of Interest

PN received research funding from Bristol Myers Squibb. The remaining authors declare that the research was conducted in the absence of any commercial or financial relationships that could be construed as a potential conflict of interest.
